# Danish translation, cross-cultural adaptation, and electronic migration of the World Endometriosis Research Foundation Endometriosis Phenome and Biobanking Harmonisation Project Endometriosis Patient Questionnaire

**DOI:** 10.3389/fgwh.2023.1102006

**Published:** 2023-03-13

**Authors:** Line Holdgaard Thomsen, Laura Emilie Vexø, Tine Henrichsen Schnack, Karina Ejgaard Hansen, Axel Forman, Dorthe Hartwell, Henriette Svarre Nielsen, Lone Hummelshoj, Mette Nyegaard, Mette Elkjær Madsen

**Affiliations:** ^1^ Department of Gynecology and Obstetrics, Odense University Hospital, Odense, Denmark; ^2^ Department of Clinical Research, University of Southern Denmark, Odense, Denmark; ^3^ Department of Gynecology, Rigshospitalet, Copenhagen University Hospital, Copenhagen, Denmark; ^4^ Department of Obstetrics and Gynecology, Copenhagen University Hospital, Hvidovre, Denmark; ^5^ Department of Public Health, Aarhus University, Aarhus, Denmark; ^6^ Department of Gynecology and Obstetrics, Aarhus University Hospital, Aarhus, Denmark; ^7^ Department of Clinical Medicine, University of Copenhagen, Copenhagen, Denmark; ^8^ World Endometriosis Research Foundation (WERF), London, United Kingdom; ^9^ Endometriosis.org, London, United Kingdom; ^10^ Department of Health Science and Technology, Aalborg University, Aalborg, Denmark

**Keywords:** endometriosis, questionnaire, Danish, EPHect EPQ, eletronic migration, translation, cross-cultural adaptation, validation

## Abstract

**Objectives:**

This study aims to translate and cross-culturally adapt the standard version of the World Endometriosis Research Foundation (WERF) EPHect Endometriosis Patient Questionnaire (EPQ) into Danish and to ensure equivalence of a Danish electronic version.

**Methods:**

The translation, cultural adaption, and electronic migration followed recommendations from the International Society for Pharmacoeconomics and Outcomes Research (ISPOR) and the Critical Path Institute. Ten women with endometriosis were enrolled for cognitive debriefing of the paper version (pEPQ) after translation and back translation. The questionnaire was then migrated into an electronic version (eEPQ) and subsequently tested for usability and measurement equivalence by five women with endometriosis.

**Results:**

Cross-cultural alterations were needed for medical terms, response options for ethnicity, the educational system, and measurement units. Thirteen questions were altered after back translation, while 21 underwent minor changes after cognitive debriefing. After testing the eEPQ, 13 questions were altered. Questions tested for measurement equivalence across the two modes of administration were found comparable. The median time-to-complete the pEPQ and eEPQ was 62 min (range: 29–110) and 63 min (range: 31–88), respectively. General comments included the questionnaire being relevant but long and repetitive.

**Conclusions:**

We find the the Danish pEPQ and eEPQ similar and comparable to the original English instrument. However, attention must be drawn to questions regarding measurement units, ethnicity, and educational systems before cross-country comparison. The Danish pEPQ and eEPQ are suitable for obtaining subjective data on women with endometriosis.

## Introduction

Endometriosis is an estrogen-dependent chronic inflammatory disease, affecting 10% of women primarily during their reproductive years (1). It is characterized by endometrium-like tissue outside the uterine cavity and is mainly associated with cyclic or chronic pelvic pain, fatigue, fertility-, bowel-, sexual- and urinary problems (2). These symptoms may affect physical, mental, and social well-being (3).

In 2013, the World Endometriosis Research Foundation (WERF) created an international collaboration to develop the Endometriosis Phenome and Biobanking Harmonization Project (EPHect) (4–7).

The purpose of EPHect is to enable large-scale, cross-centre, epidemiologically robust research into the causes of endometriosis, novel diagnostic methods, and better treatments. This is facilitated through the development of tools for detailed clinical and personal phenotyping (phenome) data to be collected from women with endometriosis and controls, as well as standard operating procedures (SOPs) for biological samples with respect to collection, transport, processing, and long-term storage (8). The WERF EPHect tools currently comprise four instruments: an endometriosis patient questionnaire (EPQ), a surgical form, and two SOPs for the collection of fluid and tissue. To date, these tools are used in 54 centres in 22 countries (9).

The EPQ is a self-administered questionnaire designed to capture anamnesis and phenotypic variations, including symptoms and health status of those with and without endometriosis. It is designed for research and should not be implemented in its full length as a clinical tool, since it is not suitable for making immediate clinical decisions. The EPQ is available in a minimum (EPQ-M) and a standard (EPQ-S) version. In the EPQ-M, sections on symptoms or characteristics during the participants' life course are omitted. Otherwise, the two versions are identical (5).

A self-administered questionnaire can measure non-quantifiable subjective information such as perception of symptoms, health, or treatment effects without interpretation by an interviewer (10). The use of Patient-Reported Outcome (PRO) questionnaires is expanding and encouraged (10–12). In addition, electronic data collection has emerged and is increasingly used in medical research (13). Electronic self-administered PROs have several benefits including automatic skip patterns, improved compliance, and reduced data management burden. Therefore, electronic PROs are claimed to be superior to paper versions (13, 14).

Combining data from non-equivalent questionnaires will result in invalid research data and measurement bias (10, 15). This emphasizes the importance of high-quality translation, validation, and cultural adaptation of questionnaires used across multiple centres (15, 16). Easy access to the description of this process is recommended (15, 17). This allows investigators to evaluate the translated questionnaires, ensuring comparable and reliable data across language versions. In addition to the original English version, the EPQ is available in 15 different languages (excluding the Danish version) (18); five of which are described in terms of their translation and cross-cultural adaptation process in varying details (19–23). It is requested by the EPHect working group that any changes made to the questionnaire are stated in any resulting publications (5). If a questionnaire is also electronically migrated, additional tests are recommended depending on the extent of alterations performed during the electronic migration process (14, 24).

This study aimed to translate and cross-culturally adapt the English paper version of the EPQ-S into Danish and to develop an electronic version while ensuring comparability between the paper and electronic mode of administration.

## Methods

The translation, cultural adaption, and electronic migration of the EPQ-S, followed the Good Practice Reports from the International Society for Pharmacoeconomics and Outcomes Research (ISPOR) and Best Practice Recommendations from the Critical Path Institute (15, 24–26). The final Danish paper version (pEPQ) was electronically migrated (eEPQ) into the secure web application “Research Electronic Data Capture” (REDCap 10.6.18-© 2021 Vanderbilt University) (27, 28).

The EPQ incorporates the McGill Pain Questionnaire and the Pain Catastrophizing Scale. These are already translated and validated into Danish and were omitted from the translation process (29, 30).

### Participants

We invited women diagnosed with endometriosis by either ultrasound, laparoscopy, or histology to complete the pEPQ and the eEPQ. The women were recruited through the Danish Endometriosis Patients Association or the Endometriosis Clinic at Rigshospitalet, Copenhagen University Hospital. Participants had to be fluent in Danish. All women provided prior verbal and written informed consent.

### Translation and cultural adaption (pEPQ)

**Figure 1:**
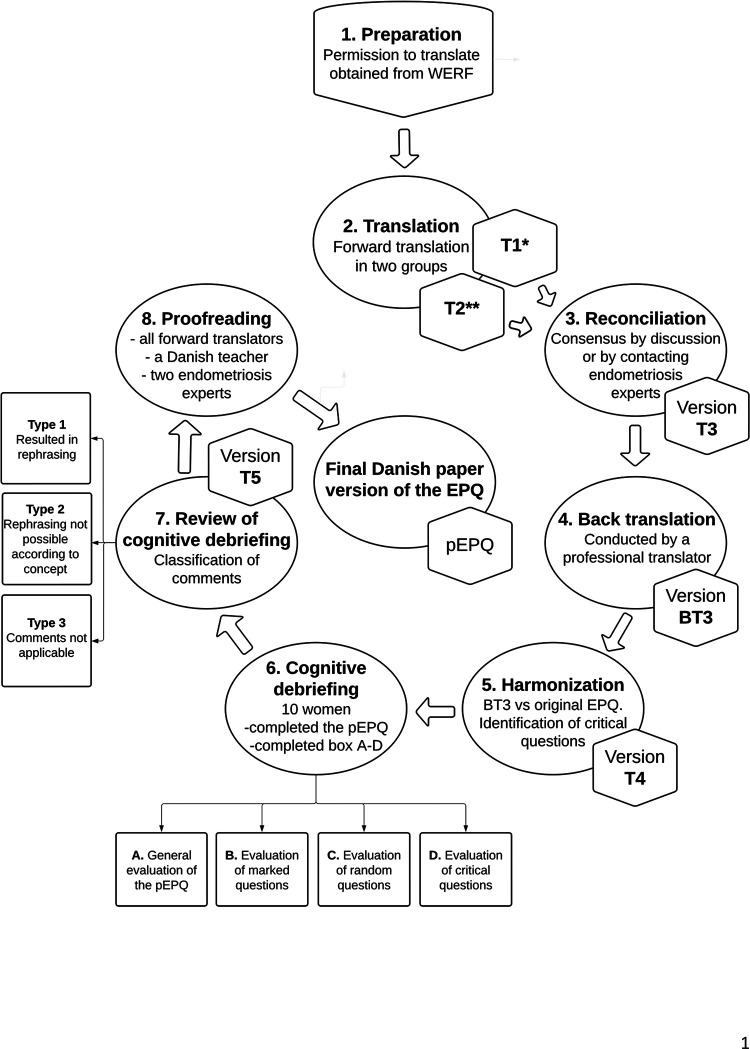
Demonstrates the translation and cultural adaptation process of the original English EPQ into Danish. *Version T1 was translated by one resident in obstetrics and gynecology and one medical student, **Version T2 was translated by one resident in obstetrics and gynecology, one associated professor in biomedicine, and one psychologist. EPQ, Endometriosis Patient Questionnaire; p-EPQ, Danish paper version of the EPQ; WERF, World Endometriosis Research Foundation. Created in Lucidchart (www.lucidchart.com).

Figure 1 illustrates the translation and cultural adaption of the English paper version into Danish.

All the translators were native Danish speakers, were fluent in English, and knew the purpose and set-up of the questionnaire. They were asked to keep the translation simple and as close to the original English version as possible (17).

Two multidisciplinary teams completed an independent translation each (T1 and T2). Representatives from each group discussed any discrepancies. Non-resolvable disagreements were settled by contacting experts in endometriosis (i.e., gynecologists specialized in endometriosis). One initial forward translation was agreed upon (T3).

### Back translation and harmonization (pEPQ)

An independent professional translator, with English as his native language and fluent in Danish, performed the back translation (BT3). The professional translator was not given any prior information on the concept of the questionnaire.

Representatives from both groups (T1 and T2) compared the back translation (BT3) to the original English EPQ. Discrepancies were evaluated and by consensus, questions were either preserved, rephrased, or back translated a second time. Questions, where the representatives debated the interpretation by a layperson, were categorized as “critical”. These questions had to be evaluated by each participant during cognitive debriefing. A medical doctor with English as his native language and fluent in Danish conducted a second back translation of relevant questions. Consensus on a preliminary version (T4) for cognitive debriefing was obtained.

### Cognitive debriefing, review, and proofreading (pEPQ)

Ten women were recruited by age, ensuring at least two in each of the following age groups: 18–29, 30–39, 40–49, and 50–60 years. All participants were asked to complete the T4 version without involving the interviewer. Participants were asked to mark phrases, figures, or questions they found inappropriate, unclear, or difficult to understand or answer. The interviewer noted time-to-complete.

After completion, the participants' initial thoughts and comments about the instrument were noted, followed by an evaluation of 1) their marked questions, 2) the “critical questions”, and 3) 5–8 randomly selected questions. The random questions were drawn using the website: www.randomresult.com. Any discrepancies between the participant´s and the intervieweŕs interpretation of a translated question led it to be rephrased in collaboration to achieve the same semantics as in the original EPQ. Extensive notes and quotes were saved for future verification. Representatives from the translation groups evaluated all comments and categorized these as either: Type 1: comments that led to rephrasing, Type 2: comments relevant for a possible second revision of the original questionnaire, and Type 3: comments not relevant for rephrasing or a second revision. These categories were based on whether the comments would improve the understanding without changing the concept of a question. Disagreements were resolved by contacting the other translators, experts in endometriosis, or the first author of the original publication (5). Lastly, it was assessed how many times each section in the questionnaire had been answered to ensure that no section had been omitted. Consensus on a single version (T5) was obtained and sent to be proofread by all translators, two experts in endometriosis, and a Danish primary school teacher. Everyone approved the final pEPQ.

### Electronic migration and determination of the extent of modifications (eEPQ)

**Figure 2:**
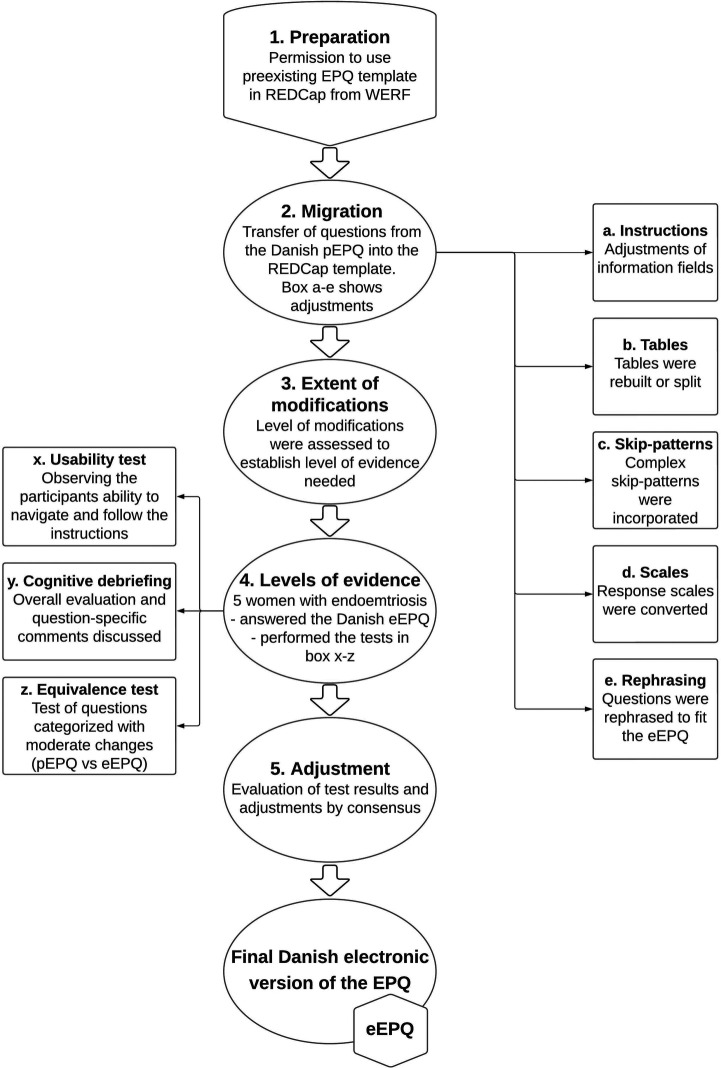
Demonstrates the steps completed to validate the electronic version of the Danish eEPQ. eEPQ, Danish electronic version of the Endometriosis Patient Questionnaire; EPQ, Endometriosis Patient Questionnaire; pEPQ, Danish paper version of the Endometriosis Patient Questionnaire; REDCap, research electronic data capture; WERF, World Endometriosis Research Foundation. Created in Lucidchart (www.lucidchart.com).

Figure 2 illustrates the migration of the pEPQ into the electronic version (eEPQ).

The first author of the original EPQ (5) provided an English REDCap template. Each question from the pEPQ was migrated to the electronic template. Semantic equal questions were given the same variable name as in the English electronic version. This enables easy extraction and aggregation of results across language versions.

In general, the format of the original paper questionnaire was kept in the electronic version, and necessary design changes were kept as close to the paper version as possible. The extent of design changes for each question was categorized as either minor, moderate, or substantial. According to these categories it has previously been recommended which tests are required to ensure comparability across modes of administration (14, 24).

### Levels of evidence and final adjustments (eEPQ)

Five women were recruited to test the eEPQ. The *usability* test was performed by asking participants to complete the eEPQ while being observed. Special attention was given to their navigation of the instrument and whether layout alterations were carried out as intended. If they experienced difficulties or doubts *during* completion, the interviewer was informed, and notes were taken. It was ensured that all sections in the questionnaire were answered by at least one participant. Time-to-complete was registered.

During *cognitive debriefing*, the participants provided an overall assessment of the questionnaire and elaborated upon any difficulties noted during completion. *Equivalence testing* was performed by having the participants answer the paper version of questions that previously had been categorized as being moderately or substantially altered during electronic migration.

### Ethics

The study was approved by the Danish Data Protection Agency (P-2021-513) and was exempted from ethical approval by the Regional Ethical Committee for the Capital Region (journal number: 19011813).

### Statistics

IBM SPSS Statistics 25.0.0.2 (IBM Corp. Released 2017. IBM SPSS Statistics for Windows, Version 25.0. Armonk, NY: IBM Corp.) was used for the statistical analysis. Outlier ranges were evaluated using the “1,5*interquartile-range” rule. The Intraclass Correlation Coefficient (ICC) was calculated to evaluate the correlation between the ratio and ordinal scale data from the two modes of administration.

## Results

**Figure 3:**
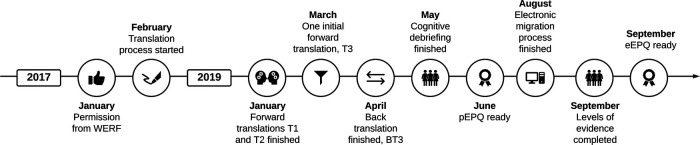
Timeline for developing the Danish paper and electronic EPQ. eEPQ, Danish electronic version of the Endometriosis Patient Questionnaire; EPQ, Endometriosis Patient Questionnaire; pEPQ, Danish paper version of the Endometriosis Patient Questionnaire; WERF, World Endometriosis Research Foundation. Created in Lucidchart (www.lucidchart.com).

The timeline is illustrated in Figure 3.

### Translation and cultural adaptation (pEPQ)

Cross-cultural adaptation difficulties were encountered regarding medical terms, commercial product names, ethnic origin, and the definition of pregnancy loss according to gestational age. The Danish translation of these questions was kept equal in concept to the original questionnaire. Response options on ethnic origin/race, major ancestry, the educational system, and measurement units (questions F4, F6, F15, and F17) had to be changed to fit Danish standards. These questions are therefore not comparable across language versions (Supplementary Appendix S1).

### Back translation and harmonization (pEPQ)

Discrepancies between the original English EPQ and BT3 resulted in 18 questions being re-evaluated. Three of these were back translated a second time. In total, 13 out of the 18 questions were altered to ensure semantic equivalence.

During harmonization, eight questions were categorized as “critical” (Supplementary Appendix S2).

### Cognitive debriefing, review, and proofreading (pEPQ)

Baseline characteristics and group distributions are presented in Table 1.

**Table 1:** Baseline characteristics including time-to-complete.

Characteristic	pEPQ (*n* = 10)	eEPQ (*n* = 5)
Age in years, median (range)	33.5 (19–53)	36 (23–43)
BMI in kg/m^2^, median (range)	25.1 (19.7–30.4)	23.6 (19.1–37.0)
Age in years at endometriosis diagnosis, median (range)	29 (17–45)^a^	30 (25–36)^a^
No. of pregnancies, number (%) 0 1 2 >3	5 (50%) 1 (10%) 3 (30%)1 (10%)	1 (20%) 0 (0%) 1 (20%) 3 (60%)
No of parities, number (%) 0 1 2 >3	5 (50%) 3 (30%) 1 (10%) 1 (10%)	1 (20%) 0 (0%) 4 (80%) 0 (0%)
Menstruation in the last 3 months, number (%)^c^	6 (60%)	2 (40%)
Pain during menstruation, number (%)^c^	10 (100%)	5 (100%)
Pain during intercourse, number (%)^c^	7 (70%)	4 (80%)
General pain in the lower abdomen, number (%)^c^	6 (60%)	3 (60%)
Hormones ever used, number (%)^c^	10 (100%)	5 (100%)
Received fertility treatment, number (%)^c^	3 (30%)	1 (20%)
Ethnicity, number (%) Caucasian Mixed ethnicity	9 (90%) 1 (10%)	5 (100%) –
Highest educational level completed, number (%) Vocational college Upper secondary school University or higher	1 (10%) 3 (30%) 6 (60%)	– – 5 (100%)
**Use computer daily, number (%)**	–	4 (80%)
**Time to complete in minutes, median (range)**	62 (29–110)	63 (31–88)
Median time to complete by age group, minutes (range) 18–29 years 30–39 years 40–49 years 50–59 years	52.5 (29–83) 42 (39–45) 87.5 (65–110) 70 (60–80)	42^b^ 54.5 (31–78) 65 (42–88) –

eEPQ, electronic version of the Endometriosis Patient Questionnaire; pEPQ, paper version of the Endometriosis Patient Questionnaire.

^a^ One missing value.

^b^ One participant in this age group.

^c^ Questions that “opens” a section in the questionnaire, if answered yes.

**Figure 4:**
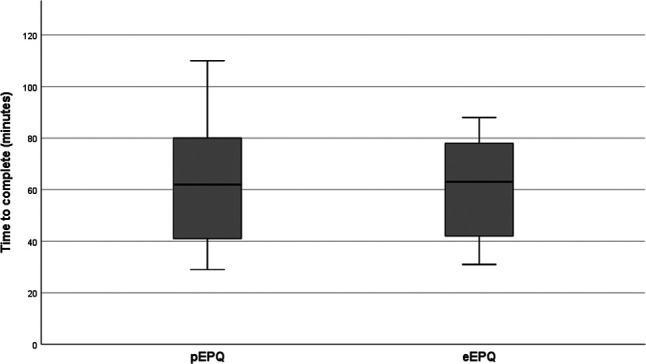
The box and whiskers plot illustrates the time taken by participants to complete the paper version (pEPQ) and the electronic version (eEPQ). eEPQ, Danish electronic version of the Endometriosis Patient Questionnaire; pEPQ, Danish paper version of the Endometriosis Patient Questionnaire.

The median time-to-complete was 62 min (range: 29–110), as illustrated in Figure 4. None of the participant's time-to-complete could be classified as outliers (data not shown). Questions that were skipped by mistake were addressed through minor layout changes or supplemental information. Each section was answered by at least three participants (Table 1).

Overall, the questionnaire was reported to be easy to read and understand (50%). However, the questionnaire was described as lengthy and time-consuming (80%), but only one participant found it too long (10%). Difficulties relating to either recall or details required were described (60%), as were a feeling of repetitiveness (40%). A lack of questions relating to pain outside the pelvic area was highlighted (40%), including discomfort in the upper abdomen, the back, and pain due to sacral nerve involvement. One woman felt questions relating to the physical and psychological side effects of medical treatment and multiple surgeries should have been included.

The participants marked 35 different questions while answering, and 50 different questions were drawn randomly. After cognitive debriefing of the marked, random, and “critical questions”, a total of 21 questions were altered based on comments categorized as Type 1 (Supplementary Appendixs S2, S3). A total of 23 comments were categorized as Type 2 and may be relevant for a possible second revision of the original questionnaire (Supplementary Appendix S4). Only alterations that did not compromise the comparison to the original English version were implemented during this phase.

### Electronic migration and determination of the extent of modifications (eEPQ)

After the electronic migration, two tables and the Numeric Rating Scale were categorized as moderately modified and assigned for equivalence testing (Supplementary Appendixs S5, 6). The design alterations of the remaining questions and tables were categorized as minor and only required usability testing and cognitive debriefing (24).

### Levels of evidence and final adjustments (eEPQ)

**Figure 5:**
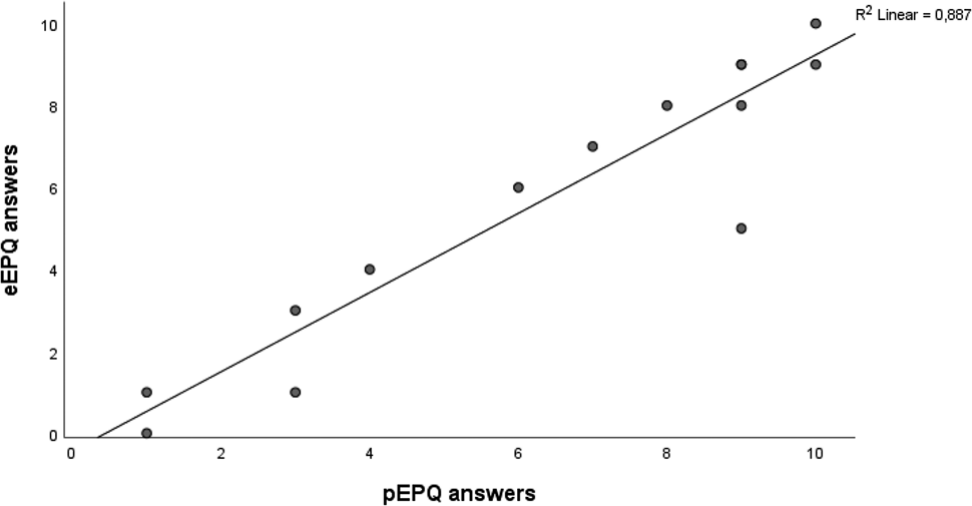
Shows the agreement of the numeric rating scale (NRS) answers across the electronic version (eEPQ) and the paper version (pEPQ). No pain is 0 and worst imaginable pain is 10. eEPQ, Danish electronic version of the Endometriosis Patient Questionnaire; pEPQ, Danish paper version of the Endometriosis Patient Questionnaire.

When testing the eEPQ's *usability*, all participants navigated the questionnaire without problems, and only minor changes were required. The median time-to-complete was 63 min (range: 31–88), and no sections were omitted (Table 1 and Figure 4). During *cognitive debriefing,* the participants reported the electronic questionnaire to be intuitive and easy to navigate. They felt confident they could answer it on their own. Everyone described the eEPQ as long and found it difficult to differentiate between the questions. They regularly had to revise previous answers to ensure they had not misinterpreted former questions. This led us to re-evaluate the eEPQ, focusing on the instructions for each question. A total of 13 questions were altered, i.e., a rephrasing of the question or response options and/or modified instructions (Supplementary Appendix S5). The participants preferred the electronic version of the tables that were *equivalence tested* (Supplementary Appendix S6). They felt it was easier to view one question at a time, instead of one large table with multiple sub-questions*.* The Numeric Rating Scale answers had an ICC of 0.96 (*P *< .001) across the two modes of administration (Figure 5). The table featuring ordinal data had an ICC of 0.74 (*P *= .026) across the two modes. A table featuring nominal data showed no difference across the two modes of administration.

## Discussion

This study is the first to translate and cross-cultural adapt the WERF EPHect EPQ into Danish. It describes the electronic migration of the questionnaire, and present patient-reported recommendations on how the questionnaire could be improved in a potential second edition.

With its detailed appendices, this study's decision-making process is relevant for international investigators in the field of translating and validating questionnaires; examples which were previously missing in the literature.

Cross-culturally adapting the questionnaire into Danish necessitated four questions to be modified in a way that compromised the possibility of direct cross-country comparison. These questions concerned heritage, measurement units, and educational level. Using terminology common to the Danish layperson should make replying easier, less confusing, and more accurate. Results from the Danish versions can easily be converted back to the corresponding English equivalent, and thus, cross-country data can be combined.

The Danish pEPQ and eEPQ were validated step by step, and each stage revealed inaccuracies that led to alterations. This illustrates the importance of a stringent methodology as was applied in this study. We recommend that none of the steps incorporated in our process should be omitted when translating, cross-culturally adapting, and electronically migrating the EPQ into other languages. However, we acknowledge that the process described in this study is time- and resource-demanding. Investigators considering to simplify or omitting any of the steps should critically evaluate the consequences in advance (15). We encourage investigators to publish or make their process of developing a new language version of the EPQ public. Transparency of such processes will allow investigators to evaluate whether data across language versions are comparable or whether these, potentially, could generate non-comparable results.

The length of the questionnaire made it impossible to evaluate all the questions with each participant during cognitive debriefing. Instead, it was necessary to incorporate adaptations by introducing random questions to reveal any potentially hidden misconceptions that may otherwise have been missed. None of the randomly drawn questions led to alterations that were not otherwise identified during cognitive debriefing. We, therefore, consider the risk of potential hidden misconceptions to be minimal. However, since some of the randomly drawn questions led to comments relevant for a second revision of the original EPQ, we still find this part of the process important.

During the electronic migration, the extent of design changes for each question was not always easy to categorize. As a precaution, all questions/tables that were suspected to be moderately modified were assigned to an equivalence test (24). This approach may explain the high correlations and confirms the high level of agreement across the two modes of administration. This result is supported by the literature stating that equivalence across administration modes is often good unless moderate/substantial changes are implemented. It also emphasizes the benefits of having patients performing cognitive debriefing of layout and formatting (14, 24). Since we implemented a total of 20 changes during this process, we confirm the importance of testing an electronic version (Supplementary Appendix S5).

Due to the incorporated skip functions, it was expected that the eEPQ would take less time to complete. Surprisingly, time-to-complete did not seem to differ between the paper and the electronic version. This could be due to unintentional selection bias, since all women testing the eEPQ were <50 years old and reported a higher educational level. However, assuming both factors serve as a proxy for increased computer skills, these would be expected to reduce time-to-complete for the electronic version, which they did not. A more likely explanation could be the observed differences in the number of pregnancies between the two groups. Participants answering the electronic version reported a higher number of pregnancies and parities (Table1). For each pregnancy, at least eight additional questions must be answered, which will increase time-to-complete. Eliminating such bias that may influence time-to-complete would require a much larger sample size and randomized inclusion.

Compared to the previously published papers, describing their translation and cross-cultural adaptation of the WERF EPHect EPQ in depth, we find our method equivalent (19, 20). This study included the lowest number of participants recommended (15). However, the comments reported in all three different studies are similar indicating that a larger number of participants likely would not have resulted in additional critical feedback. We believe that the method applied i.e., inclusion by age, evaluation of critical, marked, and random questions, including the fact that each section was evaluated by at least 9 participants in total (Table 1), compensates for the low number of participants.

Comparing the time-to-complete reported by previous published translation and validation studies of the EPQ, our study reports the highest time-to-complete (19, 20). A plausible explanation could be differences in the study design as one study tested a shorter version (EPQ-M) which expectantly led to a faster time-to-complete [36 ± 10.8 min (20)], and another study tested the questionnaire without supervision [30–60 min (19)]. Assuming answering under supervision encourages the participants to keep focus and not to cut corners, we would expect our design and method to result in an increased time-to-complete, as observed (22).

All studies that published comments evaluating the questionnaire reported it being long and/or repetitive (19, 20, 22). Despite these comments, we recommend that investigators use the full questionnaire to ensure the possibility of future collaborations with other research institutions. However, if implemented in a clinical setting, relevant questions could be selected.

Women without endometriosis were not included in our study. Since the questionnaire was not designed as a construct-specific tool, i.e., to capture change over time or to compare specific case and control groups (5), we did not find this essential for the questionnaire to be culturally valid or equal in content to the original English EPQ. Thus, whereas the questionnaire is to be used to capture differences between case and control groups, this may necessitate further validation.

During equivalence testing, participants were not randomly assigned to respond to the paper or electronic version first. This may have influenced their response regarding their preferred administration mode (24).

A considerable strength of this study was the inclusion of women with endometriosis of differing ages during cognitive debriefing of the pEPQ. It increased the variability of the participants' history with endometriosis, as well as the diversity in their approach to the questionnaire's semantics and concept (10). We find this heterogeneity to be reflected in Table 1, as well as in the overall feedback, highlighting limitations not previously reported (i.e., pain outside the pelvic area and side effects associated with treatments).

## Conclusion

The results of this study show that the Danish paper and electronic EPQ are equivalent and comparable to the original English instrument. However, special awareness is required regarding the non-equally translated questions due to cross-cultural differences. For future translations of the EPQ, we recommend that the questionnaire should be thoroughly evaluated by several translators, experts in endometriosis, and those with lived experience of endometriosis. This regards the development of both a paper and/or an electronic version of the EPQ. We recommend that patients should be informed about the purpose and length of the questionnaire to minimize any frustrations related to the time spent answering the questionnaire.

## Data Availability

The data for this study is not publicly available because it contains person-identifiable information. Anonymized data can be supplied upon request.
