# Supplementary material for: Danish translation, cross-cultural adaptation, and electronic migration of the World Endometriosis Research Foundation Endometriosis Phenome and Biobanking Harmonisation Project Endometriosis Patient Questionnaire
Source: Front Glob Womens Health. 2023 Mar 13;4:1102006. doi: 10.3389/fgwh.2023.1102006 (PMC10042231; doi:10.3389/fgwh.2023.1102006)
Supplement: Supplementary file 1 [file Datasheet1.docx]

APPENDICES 1-6

Appendix 1: Translated questions cross-culturally adapted to Danish standards 2

Appendix 2: Identified critical questions 4

Appendix 3: Overview of random and marked questions 7

Appendix 4: Type 2 comments 12

Appendix 5: Modifications added after usability testing and cognitive debriefing of the eEPQ 15

Appendix 6: Equivalence testing 17

Appendix 1: Questions that were cross-culturally adapted to Danish standards

Table of translated questions changed in the Danish EPQ due to cross-cultural differences.

| **APPENDIX 1**  **The Danish cross-culturally adapted questions** | |
| --- | --- |
| **English original version** | **Cross-culturally adapted version, shown in English** |
| F4. What is your major ancestry? (Please check one).  🞎 Scandinavian  🞎 African  🞎 Irish / Celtic / British  🞎 Asian  🞎 Other Northern European  🞎 Southern European / Mediterranean  🞎 Eastern European  🞎 Other (Please specify): ___________  🞎 South American  🞎 Don’t know  🞎 Central American or Caribbean | F4. How would you describe your ethnic origin?  🞎 Caucasian (descendent of Europeans, i.e., the majority of the population in Europe, North Amerika and Australia. Includes descendants from the Middle East and African countries north of the Sahara)  🞎 African (African countries south of the Sahara)  🞎 East Asian (descendants from countries around China, Korea, and Japan) 🞎 South Asian (descendants of countries around Pakistan, Bangladesh and India)  🞎 Latin American  🞎 Another ethnicity  🞎 Mixed ethnicity |
| F6. What is the highest level of education you have attained (with certificate)?  🞎 Primary/grade school 🞎 Lower secondary/middle school 🞎 Upper secondary/high school 🞎 Post‐secondary not university / some college or vocational school  🞎 University 🞎 Postgraduate | F6. What is the highest degree of education, that you have completed (with certificate)?  🞎 Primary School 0.-9. class 🞎 Primary School 10. class 🞎 Combined upper secondary education (general or vocational)  🞎 Vocational college 🞎 Upper secondary school (abbreviations: STX, HHX, HTX, HF)  🞎 Academy Profession program (2-2,5 years)  🞎 Professional bachelor education (3,5-4 years) 🞎 University Bachelor (3 years) 🞎 Post graduate program (a total of 5 years or more) 🞎 Ph.D. or another research degree |
| F15. During the **last 12 months**, what was your average time **per week** spent on each of the following recreational activities?  Walking or hiking outdoors (include walking to work)  Jogging (slower than 10 minutes/mile)  Running (10 minutes/mile or faster)  Bicycling (include stationary machine)  Calisthenics/aerobics/aerobic dance/rowing machine  Tennis, squash, racquetball  Lap swimming  Other aerobic recreation (e.g., lawn mowing) | F15. During the **last 12 months**, what was your average time **per week** spent on each of the following recreational activities?  Walking or hiking outdoors (include walking to/from work)  Jogging (running slower than 6 minutes/kilometer or 10 kilometers/hour)  Running (10 kilometers/hour or faster)  Bicycling (include stationary machine)  Calisthenics/aerobics/aerobic dance/rowing machine  Tennis, squash, racquetball  Lap swimming  Other aerobic recreation (e.g., lawn mowing) |
| F17. Do you drink any alcohol?  🞎No 🞎Yes  **If yes**: F17.1. During an average week, how much do you drink of each of the following?  (*Please note exact numbers, not ranges such as 1‐3*)  **Type of alcohol (serving size)**  Beer/lager/cider (half pints (284 ml)  Sherry/vermouth/port (50 ml)  Wine (175 ml) Spirits (25 ml)  Other (*Please specify)* | F17. Do you drink any alcohol?  🞎No 🞎Yes  **If yes**: F17.1. During an average week, how much do you drink of each of the following?  (*Please note exact numbers, not ranges such as 1‐3*)  **Type of alcohol (serving size)**  Beer/cider (33 cl)  Sherry/vermouth/port (1 glass of 8 cl)  Wine (1 glass of 12 cl)  Spirits (4 cl)  Other (*Please specify)* |

Appendix 2: Identified critical questions

The table shows the eight critical questions chosen for cognitive debriefing and contains concerns, considerations, and changes.

| **APPENDIX 2**  **Critical questions** | | | | | |
| --- | --- | --- | --- | --- | --- |
| **Question** | **Question**  **(original English question)** | **Our concern (after translation into Danish)** | **No misinterpretations, N_total_=10 n (%)** | **Quotes and comments** | **Rephrased after cognitive debriefing (yes/no)** |
| **A2.4** | Were your periods in the last 3 months natural or hormone‐induced (e.g., on the pill, injections, Mirena or HRT)? | Do they know what the difference between a natural period vs. a period as part of hormonal treatment is? | 9 (90%) | One participant misunderstood and described ¨natural periods¨ as the seven-days break in birth control pills during one cycle. | No |
| **A2.9** | In the last 3 months, how many days were there between the first day of one period and the first day of the next **on average**? | Can they define which days in the menstrual cycle this includes? | 10 (100%) | - | No |
| **B1** | Termination (abortion) | Do they equate these two terminologies? | 9 (90%) | One participant who misunderstood and described ‘termination’ as a spontaneous abortion | No |
| **C14** | During the time in your life when your pelvic pain during your period was at its worst, were you taking any medication to help alleviate the pain? | A. What would they answer if they took hormones for other reason(s) than pelvic pain?  B. What would they answer if paracetamol was prescribed by a doctor? Would they choose ´as prescription´ or ´over the counter´? * | A. 7 (70%)  B. 8 (80%) | A. Three participants did not answer the question correctly and chose “Yes, hormones, but pain was not alleviated”, even though hormones were not prescribed for pain relief.  B. The two participants who did not understand the question correctly and categorized the prescription of many paracetamol tablets as a prescription drug. | A.+ B. Yes  We specified the definition of prescription painkillers and where to mark if you took hormones for other reasons than pain. C4, C32 and C40 adopted the same changes. |
| **C23** | **In the last 12 months**, was there a time of the month in which vaginal intercourse/penetration was more painful than at other times? | Do they understand the question? | 10 (100%) | 5/10 (50%) described it as confusing, that there were no response options to question 23, and that they had to go straight to question 23.1. However, no one answered wrong.  Two lacked an “I don´t know” option. | No. |
| **C29** | Have you ever experienced pelvic pain in general? **Do not count:** pain caused by menstrual cramps, intercourse, surgery, pregnancy, childbirth, sports‐related or other injury, food poisoning, or stomach flu. | A.” Do not count” is defined twice (in the introduction to this section and as a part of question C29). We wondered if it had impacted their understanding of the question?  B. Which types of pain is the question referring to? | A. 10 (100%)  B. 6 (60%) | A. It did not impair their understanding of the question, however two found it a bit confusing.  B. Six of the women defined the type of pain to be both chronic and sudden/unexpected pain (we consider both definitions to be correct).  Four women did not know which type of pain the questions referred to; of these, one defined it as pain before menarche. | A. Yes. The second “do not count” was rephrased to “Remember not to count”  B. Yes. One adverb and the grammar were altered to elaborate which type of pain the question was referring to. |
| **C31** | E: Approximately how long in total did you have this pain for **in the last 3 months**? | Do they combine/sum up the pain episodes? | 9 (90%) | Only one misunderstood the question as “how often”.  Three found if confusing that the response options were per month, week, or day, and would like “on average” added. | Yes. The phrase “on average” was added |
| **D1.1** | E: What type(s) of cancer (primary location) have you been diagnosed with, and when were you first diagnosed? | What does “primary location” mean? | 7 (70%) | Three did not redefine the question correctly; one would also count metastasis to a secondary organ at diagnosis as the primary location; one did not equate “type of cancer” to “primary location”; and one defined “type of cancer” as slow vs aggressive cancer. | Yes. “i.e.,” was added and “primary location” was replaced with “in which organ did the cancer begin” |

*In Denmark it is possible to buy paracetamol over the counter in packages of 10 tablets of 500 mg each. A larger number of tablets *and/or* tablets containing 665 mg must be prescribed by a medical doctor.

Appendix 3: Overview of random and marked questions

Full list of the random and marked questions including categorization of comments

| **APPENDIX 3**  **Overview of random and marked questions** | | | | | | |
| --- | --- | --- | --- | --- | --- | --- |
| **Question** | **Random drawn by n participants (n)** | **Marked**  **by n participants (n)** | **Comment category*** | | | **Short description of changes** |
|  |  |  | **Type 1** | **Type 2** | **Type 3** |  |
| **A1** | - | M (1) | - | - | X (1) |  |
| **A2** | R (1) | M (1) | - | X (1) | X (1) |  |
| **A2.5** | - | M (1) | X (1) | - | - | The acronym LMP was deleted |
| **A2.6** | R (2) | M (1) | - | X (3) | - |  |
| **A2.7** | R (1) | - | - | - | X (1) |  |
| **A2.8** | R (1) | - | - | X (1) | - |  |
| **A3** | R (1) | - | - | X (1) | **-** |  |
| **A4** | R (1) | M (6) | - | X (3) | X (4) |  |
| **A5** | R (1) | - | - | - | X (1) |  |
| **A6** | R (1) | M (1) | - | X (1) | X (1) |  |
| **B1** | - | M (2) | X (1) | - | X (1) | Added:  “→ go to B2” after the box labeled “No” |
| **B2** | R (2) | M (1) | X (1) | - | X (2) | Deleted ”more than” |
| **B3** | - | M (3) | - | X (3) | - |  |
| **B4** | R (1) | M (1) | - | X (1) | X (1) |  |
| **C1** | R (1) | M (2) | - | X (2) | X (1) |  |
| **C2** | - | M (3) | - | X (3) | - |  |
| **C3** | R (2) | - | - | - | X (2) |  |
| **C4** | R (2) | - | X (2) | - | - | Prescription drugs was specified as in C14 |
| **C5** | R (2) | M (1) | - | X (2) | X (1) |  |
| **C6** | R (3) | - | - | - | X (3) |  |
| **C7** | R (1) | - | - | - | X (1) |  |
| **C11** | R (2) | - | - | - | X (2) |  |
| **C12** | - | M (2) | - | X (1) | X (1) |  |
| **C13** | - | M (1) | - | - | X (1) |  |
| **C14**** | R (1) | - | - | - | - | Alterations are described in Appendix 2 |
| **C15** | R (1) | M (2) | - | X (3) | - |  |
| **C16** | R (1) | - | - | - | X (1) |  |
| **C20** | R (1) | - | - | - | X (1) |  |
| **C24** | R (1) | - | - | - | X (1) |  |
| **C27** | - | M (1) | - | X (1) | - |  |
| **C28** | - | M (4) | - | X (4) | - |  |
| **C29.2** | R (1) | - | - | - | X (1) |  |
| **C30** | R (1) | M (1) | - | - | X (2) |  |
| **C32** | R (1) | - | X (1) | - | - | Prescription drugs and hormones were specified as in C14 |
| **C33** | R (1) | - | - | - | X (1) |  |
| **C34** | R (1) | - | - | - | X (1) |  |
| **C33-C37** | - | M (1) | X (1) | - | - | Added: “lower abdominal pain” |
| **C35** | - | M (1) | - | - | X (1) |  |
| **C37** | R (1) | - | - | - | X (1) |  |
| **C38** | R (1) | M (1) | - | X (1) | X (1) |  |
| **C39** | R (1) | - | - | - | X (1) |  |
| **C40** | R (2) | - | X (2) | - | - | Prescription drugs was specified as in C14 |
| **C41** | - | M (4) | X (1) | X (1) | X (2) | Added:  “please choose all relevant response options” |
| **C43** | R(1) | M (2) | - | X (2) | X (1) |  |
| **D2** | - | M (1) | X (1) | - | - | We changed ”other” to “other diseases” |
| **D3** | R (1) | - | - | - | X (1) |  |
| **D3.1** | R (2) | - | - | - | X (2) |  |
| **D3.2** | R (1) | - | - | - | X (1) |  |
| **D4** | - | M (3) | X (3) | - | - | Added:  “define type of surgery by the numbers below” and “other abdominal surgery than described above” |
| **D5** | R (2) | - | - | - | X (2) |  |
| **D6** | R (2) | - | - | - | X (2) |  |
| **D8.1** | R (1) | M (2) | X (3) | - | - | It was specified that it was how their endometriosis was diagnosed |
| **D8.2** | - | M (2) | X (1) | - | X (1) | It was specified that it was complete and/or partly removal of endometriosis |
| **D8.3** | R (1) | M (1) | - | - | X (2) |  |
| **D8.4** | R (1) | - | - | - | X (1) |  |
| **D9** | R (3) | - | - | - | X (3) |  |
| **D10** | R (2) | M (2) | - | X (2) | X (2) |  |
| **E1** | R (2) | M (3) | X (2) | X (2) | X (1) | It was emphasized that it was “regular” use of painkillers |
| **E2** | R (1) | M (1) | - | X (1) | X (1) |  |
| **F2** | R (2) | - | - | - | X (2) |  |
| **F4** | - | M (1) | - | - | X (1) |  |
| **F6** | - | M (2) | - | - | X (2) |  |
| **F9** | R (1) | - | - | - | X (1) |  |
| **F10** | R (2) | - | - | - | X (2) |  |
| **F12** | R (2) | - | - | - | X (2) |  |
| **F14** | - | M (1) | - | - | X (1) |  |
| **F15** | R (1) | - | - | X (1) | - |  |
| **F16** | R (2) | - | - | - | X (2) |  |

*Type 1: These comments resulted in the alteration of the Danish translated question, Type 2: These comments were found relevant, but the suggested rewordings were not possible, since it would change the concept of the question making it non-comparable to the original English version. Type 3: There was no misunderstanding of the question, and the comments were not relevant for rephrasing. **This question was also evaluated as part of the eight critical questions, please see Appendix 2.

Appendix 4: Type 2 comments

List of comments (Type 2) that could be relevant for a possible second revision of the original EPQ questionnaire.

These suggestions are based on patient-reported feedback from the Danish translation and cross-cultural adaptation process, therefor not all comments may be applicable across languages and cultures.

| **APPENDIX 4**  **Type 2 comments** | | |
| --- | --- | --- |
| **Question** | **Number of times the question was randomly drawn (R(n)) or marked by a participant (M(n))** | **Comment(s)** |
| **A2** | R (1)/ M (1) | Participants were confused about how to answer, if prescribed contraceptives continuously, with only few breaks/periods a year. They lacked an option, i.e., “irregular (due to hormones prescribed by my doctor)”. One participant ticked several boxes. |
| **A2.6** | R (2)/ M (1) | Same comments as A2. |
| **A2.8** | R (1) | Heavy bleeding is defined by “cloths/flooding” in A3 but not in A2.8. Participants asked if the definition of “heavy” in A2.8 and A3 were same? |
| **A3** | R (1) | The introduction was described as long and difficult to remember while answering the questions. |
| **A4** | R (1)/ M (6) | Two of the participants lacked an option: Hormones in relation to fertility treatment.  One raised the same problem as described in A2 and A2.6. |
| **A6** | R (1)/ M (1) | One checked “other” and wrote endometriosis since she was already diagnosed. However, then she did not answer A6.1 and A6.2. |
| **B3** | M (3) | One participant lacked the option; “No, because we knew we couldn’t get pregnant”.  Another participant was examined for infertility, but initially no cause was found. Later she was diagnosed with endometriosis. She did not know which response option she should choose in B3.1.  A third found it strange that she had to answer this, since she had never tried to get pregnant. |
| **B4** | R (1)/ M (1) | One participant found it strange that she had to answer this, since she had never tried to get pregnant. |
| **C1 and C34** | R (1)/ M (2) | Two participants did not know what kind of pain the question was referring to: endometriosis-related pain or pain not related to endometriosis? |
| **C2** | M (3) | Three participants noted that the response options did not match the questions. They would also like to choose more than one response option. |
| **C5** | R (2)/ M (1) | Two participants lacked an option called “Yes, but not to alleviate pain”. |
| **C12 and**  **C38** | M (2)  R (1)/ M (1) | One participant wrote an age interval. We decided to accommodate the electronic version to fit this (Appendix 5). |
| **C15** | R (1)/ M (2) | One participant would like to choose more than one response option per question.  The NRS scale incorporated in the table confused several, it was not clear whether the scale was part of the table. However, changing this would require more than simple or moderate layout changes. |
| **C27** | M (1) | The participants expressed doubt whether the question should be answered as “during” or “24 hours after” intercourse. |
| **C28** | M (4) | One participant lacked an “I don’t know” option for question no. 4  Three were confused regarding the NRS scale. Please see description of question C15. |
| **C41** | M (4) | Again, confusion regarding the NRS scale; please see description of question C15. |
| **C43** | R (1)/ M (2) | One participant expressed doubt about the type of pain the question was referring to, since she had been pregnant within the last 12 months.  One would like to choose more than one response option. |
| **D10** | R (2)/ M (2) | Two participants found it strange that daughters were not included in this question.  One participant would like “I do not have a sister” as the first option. |
| **E1** | R (2) / M (3) | Three found it difficult to answer due to recall difficulties or lack of knowledge regarding the medicine described in the table. |
| **E2** | R (1)/ M (1) | A participant did not know whether prophylactic calcium and vitamin D was defined as medicine for osteoporosis? |
| **F15** | R (1) | It was difficult to answer for one participant due to a recent pregnancy. |

Abbreviations: NRS; Numerical Rating Scale.

Appendix 5: Modifications added after usability testing and cognitive debriefing of the eEPQ

Description of changes made to the eEPQ after usability testing and cognitive debriefing.

| **APPENDIX 5**  **Modifications added after usability testing and cognitive debriefing of the eEPQ** | | | | |
| --- | --- | --- | --- | --- |
| **Question** | **1. Information field** | **2.**  **Question** | **3.**  **Response options** | **Short description of modifications** |
| **A2.8** | X | X | X | 1 & 2. Clarifying text was added due to removed heading  3. The illustration swapped places with the response options since ¾ misunderstood how to answer the question (they thought they should touch the illustration) |
| **A2.9** | - | - | X | 3. The last response option “too irregular to estimate” was moved to the top of the menu to avoid scrolling |
| **A3** | X | X | - | 1. Underlining was added in the information field  2. Clarifying text was added in the eEPQ and pEPQ |
| **A4** | X | - | X | 1. Text added, and redundant text removed  3. The response options were rephrased making it possible to choose if hormones had been used several times, including details on the different periods |
| **B1** | X | - | X | 1. Clarifying text was added  3. We added underlining to one response option and added extra response options (e.g., “had no complications”) |
| **C12** | - | - | X | 3. We made it possible to mark a range of years instead of only one specific year |
| **C15** | X | - | - | 1. Underlining was added |
| **C26** | - | - | X | 1. We made it possible to mark an age-range (since participants did this in the pEPQ) |
| **C28** | - | - | X | 3. We added a response option with identical wording to the revised C23 (Appendix 6) |
| **C30** | - | X | - | 2. Clarifying text was added due to removed heading |
| **C32** | - | X | - | 2. Clarifying text was added due to removed heading |
| **C41** | X | X | X | 1. Underlining was added  2. Clarifying text was added in a sub-question due to removed heading  3. We added one response option in a sub-question using identical wording to the revised C23 (Appendix 6) |
| **E1** | X | - | - | 1. Underlining was added |
| ***In total*** | ***7*** | ***5*** | ***8*** |  |

Appendix 6: Equivalence testing

The questions chosen for equivalence testing and our considerations.

The questions needing equivalence testing were several large tables that consisted of many sub-questions, e.g., table C23 consisted of 4-8 sub-questions.

| **APPENDIX 6**  **Equivalence testing** | | |
| --- | --- | --- |
| **Question** | **Number of**  **sub-questions tested** | **Reason for equivalence test** |
|  |  |  |
| **C8** | 1 | The exact format of the NRS* could not be transferred to the eEPQ. The numerical scale was horizontal, but the text defining “no pain” (0) and “worst imaginable pain” (10) could not be incorporated on the numerical scale. This definition was described in the information field instead. |
| **C15**  **(table)** | 8  (including two NRS sub-questions) | Three tables in the pEPQ (C15, C28, and C41) had the same layout and could not be transferred to the eEPQ in its original format. C15 was chosen for equivalence testing.  Participants were asked to answer the column of questions regarding their current age-range in the pEPQ. |
| **C23**  **(table)** | 4-8 | The layout could not be transferred to the eEPQ and it was necessary to rephrase each response option. An extra response option was added to ensure that the content was the same as in the pEPQ. |
| **C27** | 1 | Same reason as described in question C8. |

*NRS: Numerical Rating Scale. A scale to rate pain from 0 to 10.
